# Possible Role of Butyrylcholinesterase in Fat Loss and Decreases in Inflammatory Levels in Patients with Multiple Sclerosis after Treatment with Epigallocatechin Gallate and Coconut Oil: A Pilot Study

**DOI:** 10.3390/nu13093230

**Published:** 2021-09-16

**Authors:** Jose Enrique de la Rubia Ortí, Jose Luis Platero, Iván Hu Yang, Jose Joaquin Ceron, Asta Tvarijonaviciute, Pablo Selvi Sabater, María Benlloch, David Sancho-Cantus, Sandra Sancho

**Affiliations:** 1 Department of Nursing, Catholic University San Vicente Mártir, 46001 Valencia, Spain; joseenrique.delarubi@ucv.es (J.E.d.l.R.O.); ivan.hu@mail.ucv.es (I.H.Y.); sandra.sancho@ucv.es (S.S.); 2 Doctoral Degree School, Catholic University of Valencia, 46001 Valencia, Spain; joseluis.platero@mail.ucv.es; 3 Interdisciplinary Laboratory of Clinical Analysis, Campus of Excellence Mare Nostrum, University of Murcia, 30100 Murcia, Spain; jjceron@um.es (J.J.C.); asta@um.es (A.T.); 4 Hospital de La Plana, 12540 Villareal, Spain; selvi_pab@gva.es

**Keywords:** multiple sclerosis, EGCG, β-hydroxybutyrate, butyrylcholinesterase, inflammation

## Abstract

(1) Background. Multiple sclerosis (MS) is characterised by the loss of muscle throughout the course of the disease, which in many cases is accompanied by obesity and related to inflammation. Nonetheless, consuming epigallocatechin gallate (EGCG) and ketone bodies (especially β-hydroxybutyrate (βHB)) produced after metabolising coconut oil, have exhibited anti-inflammatory effects and a decrease in body fat. In addition, butyrylcholinesterase (BuChE), seems to be related to the pathogenesis of the disease associated with inflammation, and serum concentrations have been related to lipid metabolism. Objective. The aim of the study was to determine the role of BuChE in the changes caused after treatment with EGCG and ketone bodies on the levels of body fat and inflammation state in MS patients. (2) Methods. A pilot study was conducted for 4 months with 51 MS patients who were randomly divided into an intervention group and a control group. The intervention group received 800 mg of EGCG and 60 mL of coconut oil, and the control group was prescribed a placebo. Fat percentage and concentrations of the butyrylcholinesterase enzyme (BuChE), paraoxonase 1 (PON1) activity, triglycerides, interleukin 6 (IL-6), albumin and βHB in serum were measured. (3) Results. The intervention group exhibited significant decreases in IL-6 and fat percentage and significant increases in BuChE, βHB, PON1, albumin and functional capacity (determined by the Expanded Disability Status Scale (EDSS)). On the other hand, the control group only exhibited a decrease in IL-6. After the intervention, BuChE was positively correlated with the activity of PON1, fat percentage and triglycerides in the intervention group, whereas these correlations were not observed in the control group (4). Conclusions. BuChE seems to have an important role in lipolytic activity and the inflammation state in MS patients, evidenced after administering EGCG and coconut oil as a βHB source.

## 1. Introduction

Multiple sclerosis (MS) is an autoimmune disease that produces demyelination on the sheath of neurons. There are different types of the disease, although the majority are relapsing–remitting multiple sclerosis (RRMS) that can evolve into secondary progressive multiple sclerosis (SPMS) [1,2,3]. Notably, patients, particular males with late-onset relapsing–remitting multiple sclerosis (LORRMS), in other words, men over 40 years of age, reach a severe level of disability much more quickly than those with young-onset relapsing–remitting multiple sclerosis (YORRMS), i.e., between 18 and 40 years of age [4]. Furthermore, there are no differences in the evolution of the disease for patients with LORRMS regarding the first-line treatments used (oral or injectable) [5]. The main characteristic is chronic inflammation that causes a gradual increase in oxidative stress [6,7,8], which contributes to increased inflammation demyelination and neurodegeneration [7,8].

There are several physical consequences, including anthropometric changes in patients as the disease progresses, characterised by the loss of lean mass [9,10]. In addition, the loss of muscle is directly associated with an increase in body fat in MS patients [11]. This also causes a rise in inflammation [12], resulting in a loss in respiratory capacity and fatigue [13]; therefore, the patient’s disability and the progression of the disease are increased [14]. In addition, an increase in body fat is associated with a greater expression of the butyrylcholinesterase enzyme (BuChE) in blood serum [15].

BuChE is related to the pathogenesis of MS. In the central nervous system (CNS), it is mainly expressed in white matter [16] and is responsible for hydrolysing acetylcholine (ACh) and generating demyelination [17]. This process leads to interrupting neurotransmission. It also acts as a mediator and internal regulator for autoimmune interactions, modulating immune activity. In this sense, a rise in BuChE activity in active MS lesions could contribute to proinflammatory immune responses [18]. The greater the level of BuChE, the greater the decompaction of myelin [19]. Therefore, BuChE seems to be associated with an increase in inflammation which is characteristic of MS, and its production could be linked to specific inflammation markers, among which interleukin 6 (IL-6) is found [20]. In this sense, it has already been possible to establish a relationship between both molecules in another pathophysiological situation of an inflammatory nature [21]. ACh is an anti-inflammatory molecule, and when the levels of BuChE increase, ACh decreases [22]. This leads to a rise in IL-6 levels due to the lack of control of negative feedback from ACh [23].

**Figure 1:**
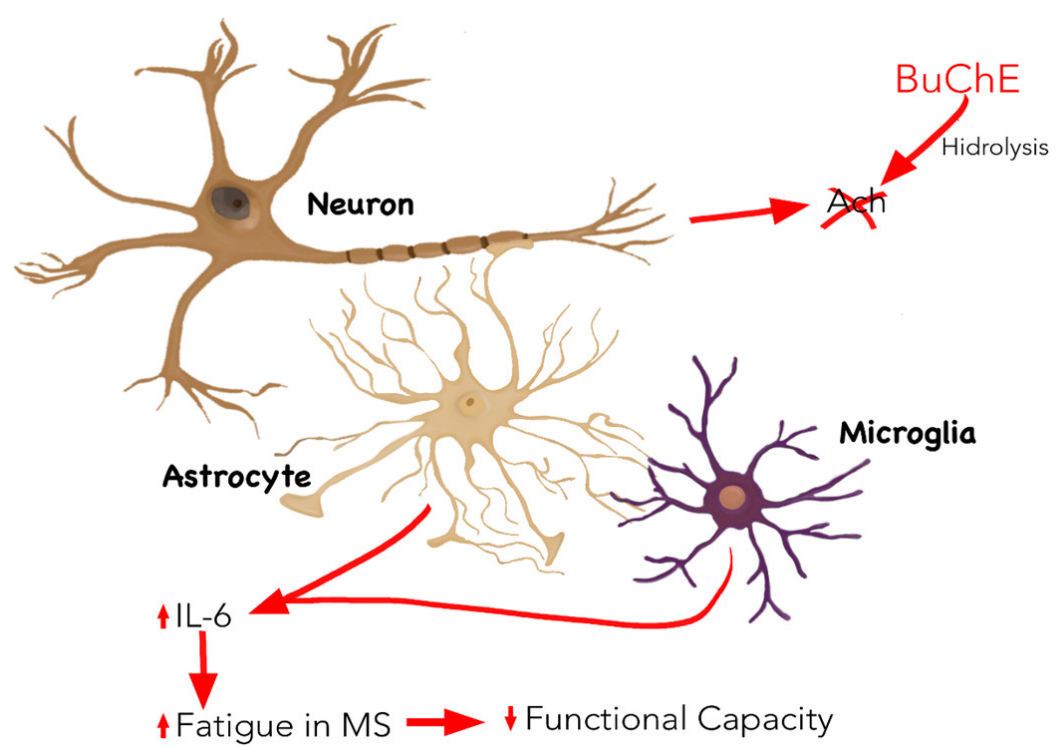
Inflammatory mechanism linked to MS in which BuChE is involved. BuChE is mainly expressed by neurons and hydrolysed ACh. An increase in BuChE activity, in particular, increases IL-6 production by decreasing ACh levels, which is associated with the appearance of fatigue, characteristic of MS, and an increase in functional disability. Abbreviations: Ach: acetylcholine ester; BuChE: butyrylcholinesterase; IL-6: interleukin 6; MS: multiple sclerosis.

In addition, and specifically in MS, the levels of IL-6 are pathologically associated with fatigue, which is characteristic of the disease, in contrast to other elements of the immune system, such as T cells and other peripheral markers of inflammation, for instance, C-reactive protein (CRP), erythrocyte sedimentation rate, or soluble forms of intercellular adhesion molecule-1 [24]. Furthermore, fatigue depends on the relapse and remission phases, which are directly related to fluctuations in IL-6 [25,26]. Finally, fatigue is also related to functional disability in these patients [27], which could explain the negative correlation between the activity of BuChE and loss of functionality in MS [28]. (Figure 1).

Thus, an increase in BuChE on a central level could play a role by producing demyelination [29] and could be a marker of the inflammation produced in the disease related to its progression. In this sense, studies of the disease with animal models (such as in experimental autoimmune encephalomyelitis) have reported greater inflammation and development of the disease in relation to a higher expression of BuChE in the CNS [30].

However, the physiological function of plasma cholinesterase on a peripheral level is not well known. On the one hand, the highest expression of the enzyme in the CNS in animal models could be correlated with levels of BuChE in the blood, because the levels in this type of sample are strongly correlated with the activity of the Ach-synthesising enzyme, choline-acetyltransferase; therefore, deregulation due to an increase in BuChE could indicate a level of inflammation at a central level [31]. However, on the other hand, BuChE has been related to lipid metabolism; thus, this activity is linked to levels of lipoproteins in plasma [32,33]. It is for this reason that BuChE could represent different roles, one of which is cholinergic signalling in states of inflammation in MS, and the other is metabolising fats linked to inflammation in pathologies, such as metabolic syndrome [34].

**Figure 2:**
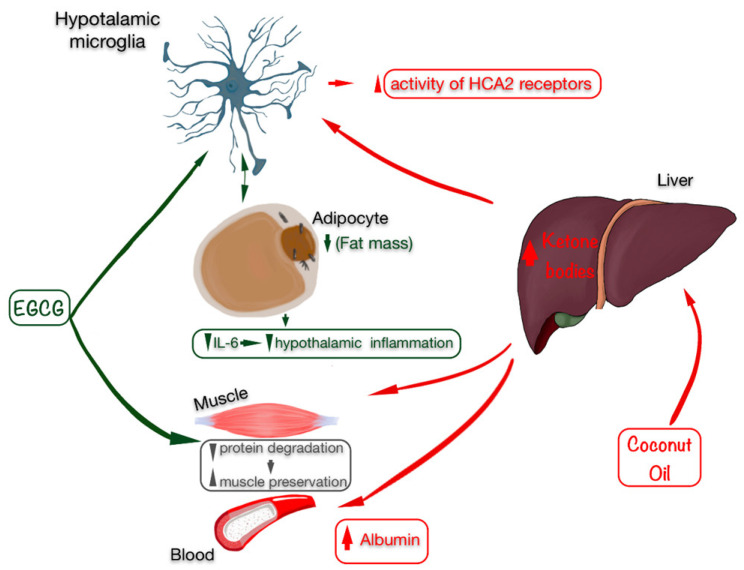
Beneficial effects of EGCG (marked in green) and coconut oil as a source of ketonic bodies (marked in red) on anthropometry by preserving skeletal muscle, improving the state of inflammation with an increase in albumin levels, and especially by decreasing fat mass, which is related to lower inflammation in the hypothalamus (microglia) and in the blood, decreasing IL-6 levels. Abbreviations: EGCG: epigallocatechin gallate; IL-6: interleukin 6.

Taking into account the relationship of body fat with the inflammatory nature of the disease, a therapeutic alternative to decrease these fat percentages could be to administer epigallocatechin gallate (EGCG), which is especially efficient to treat autoimmune diseases such as MS due to its high neuroprotective capacity [35]. As seen in animal models, EGCG eliminates the accumulation of lipids in rodents that follow a high-fat diet. As a result, proinflammatory cytokines are released, among which IL-6 is found, thereby decreasing hypothalamic inflammation induced by a high fat content [36]. In addition, along with the loss of fat, a supplementation of EGCG also manages to preserve muscles in sarcopenic rats [37], which impacts inflammation markers such as albumin (Figure 2). This increase is related to anthropometric improvements and decreased inflammation markers, such as IL-6 [38].

EGCG activity could be supplemented with the action of ketone bodies, obtained through hepatic beta-oxidation, because they have shown improvements in inflammatory markers [39]. Particularly in MS, the ketone body β-hydroxybutyrate (βHB) activates HCA2 receptors expressed by neuroinflammatory cells, reducing neuroinflammation [40] and exhibiting a neuroprotective effect [41]. Regarding the nutrients capable of providing higher levels of ketone bodies in the blood, those with high levels of medium-chain triglycerides (MCTs) stand out, with coconut oil possibly having the highest amount of MCTs, due to the high percentage of medium-chain fatty acids, such as caprylic acid, capric acid and lauric acid [42]. In addition, ketone bodies, especially βHB, have pleiotropic effects that regulate inflammation and oxidative stress and exhibit anti-catabolic effects in human skeletal muscle [43]. Furthermore, body fat mass is decreased [44].

Anthropometric improvements with fat loss can be associated with the improvement of oxidative stress determined by markers such as paraoxonase 1 activity (PON1). PON1 is related to lipid metabolism [45], inhibiting the oxidation of low-density lipoproteins (LDL), thus avoiding the production of cytokines, being especially efficient to establish metabolic improvements [46].

Based on the above, the aim of this study was to determine the role of BuChE in the changes induced after treatment with EGCG and ketone bodies on the levels of body fat associated with muscle loss and related to the inflammation state in MS patients.

## 2. Materials and Methods

### 2.1. Design

A pilot, descriptive, quantitative and experimental study was conducted. The Clinical Trial ID for this study is NCT03740295.

### 2.2. Patients

**Figure 3:**
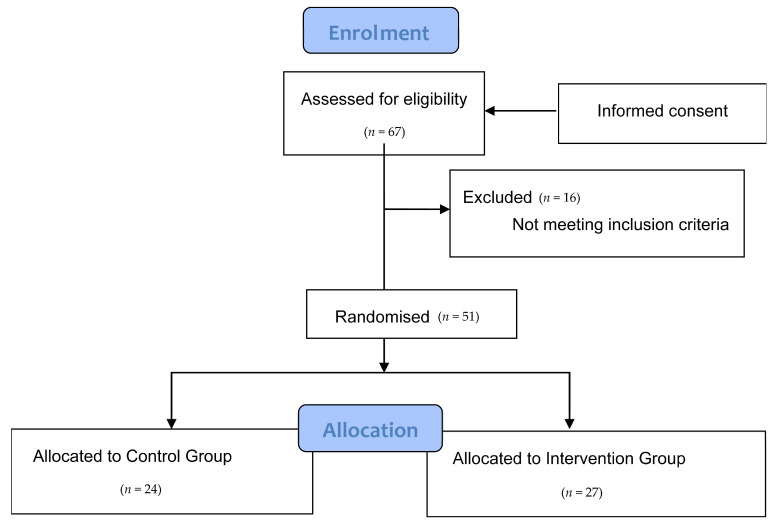
Consort flow diagram.

The participants in the study were MS patients who were affiliated members of different MS associations in the Region of Valencia. For this purpose, we previously contacted the management of each centre in order to explain the project, and they sent the information to patients of their associations. A total of 67 patients showed interest in participating, after being diagnosed with the disease by means of the McDonald test conducted by neurologists, which aims to determine that there has been damage in the CNS over time (there must be evidence that damage was caused at different times) and in space (evidence that there are lesions in at least two different parts of the CNS) based on clinical, radiographic and laboratory criteria; the measure shows great sensibility and specificity, making it a reliable tool to diagnose MS [47]. The following inclusion criteria were applied to these patients: patients over 18 years of age, diagnosed with RRMS or SPMS at least one year prior, treated with glatiramer acetate and interferon beta, and to not have had a relapse in the last 6 months. The exclusion criteria included: pregnant or breastfeeding women, patients with dementia, evidence of alcohol or drug abuse, heart disease, patients with kidney conditions with creatinine levels two times higher than normal markers, patients taking antidepressants, patients with metabolic diseases, or MS patients who were included in other studies. Once these criteria were applied, the final sample included 51 patients (Figure 3).

### 2.3. Procedure

Once the sample was obtained, the patients and their families received detailed information on the study, both verbally and with a written information sheet. Patients meeting the inclusion criteria also signed an informed consent form. Before the intervention, they registered their solid and liquid intake from the previous 7 days. A personalised isocaloric diet was tailored to them by taking into account each patient’s pathophysiology, and in accordance with the information gathered from participants and after a personal interview to discover their food habits. Diets were designed using the “Easydiet Programa de Gestión de la Consulta^®^”, software (Spanish Academy of Nutrition and Dietetics, Pamplona, Spain) where the anthropometric characteristics of each participant and their diseases were introduced in order to establish the caloric, macronutrient and micronutrient needs.

### 2.4. Intervention

Once the selection criteria had been applied, a final sample of 51 MS patients was obtained. They were randomly divided into the intervention group (27 patients) and the control group (24 patients). Randomisation to either group was performed without stratification, by drawing consecutively numbered sealed envelopes. The intervention group received an isocaloric diet for 4 months (adapted to the individual characteristics of each patient and divided into 5 meals a day: breakfast, mid-morning snack, lunch, afternoon snack and dinner) enriched with 60 mL of extra-virgin coconut oil divided into 2 equal intakes (30 mL for breakfast and 30 mL for lunch, providing them a calibrated syringe and requiring them to administer the content directly into their mouth), and supplemented with 800 mg of EGCG administered in two capsules of 400 mg to be taken twice a day (one capsule in the morning and another in the afternoon). On the other hand, the control group followed the same isocaloric diet as the intervention group for the same 4 months, except for coconut oil, as well as administering a placebo (capsules containing microcrystalline cellulose, matching in size and colour). They followed the same instructions as the intervention group. The basal diet for all subjects included the following percentage distributions of the 3 main macronutrients with respect to the total caloric value: 20% proteins, 40% carbohydrates and 40% Mediterranean lipids not rich in medium-chain fatty acids. This guaranteed that participants in the control group did not enter ketosis, because the percentages and the amounts of macronutrients were not characteristic of a ketogenic diet [48,49]; neither was the composition of lipids that made up said 40%. However, ketosis in the intervention group was foreseeable, because the highest percentage of 40% of lipids was from coconut oil rich in medium-chain fatty acids, representing 91.84% saturated fatty acids, 6.23% monounsaturated fatty acids and 1.93% polyunsaturated fatty acids, which were included in the isocaloric diet and adapted to an adult’s nutritional requirements. In terms of catechins, nutrients rich in polyphenols, especially tea and coffee, were avoided when designing the basal diet for both groups. This diet was characterised for being balanced, varied, and with sufficient calories, by providing adequate food portions divided into 5 daily intakes. The consumption of proteins with a high biological value of animal origin such as fresh fish, eggs and dairy products (milk, yoghurt and cheese) were encouraged, to the detriment of meat and meat-based products.

### 2.5. Measurements

Fat mass percentage: In order to determine the fat mass percentage of the patients, the following measurements were taken: weight, size, skin folds and body perimeters and diameters, using the Faulkner method, taking into account the protocol established by The International Society for the Advancement of Kinanthropometry (ISAK). Furthermore, an ISAK level 2 certified anthropometrist took the measurements [50]. The equipment used for these measurements were: a portable clinical scale, SECA model, with a 150–200 kg capacity and 100 g precision; a stadiometer, model SECA 220 Hamburg, Germany, with 0.1 cm precision; a mechanical skinfold calliper, model Holtain LTD Crymych, UK, with 0.2 mm precision and measurement range from 0 to 48 mm; a dermographic pencil; a metal, inextensible and narrow anthropometric tape, model Lufkin W606PM with 0.2 mm precision; and a bicondylar pachymeter to measure the diameter of small bones, model Holtain, with 1 mm precision and measuring range from 0 to 48 mm [51].

Blood test and marker analysis: A blood test was carried out on all patients at 11 a.m. on an empty stomach; then, the serum was separated from the plasma after centrifuging the samples. BuChE concentration was measured by means of a method previously reported by Tecles et al. [52]. IL-6 was measured with the ELISA technique (R&D Systems). βHB levels were measured with a commercial kit (Randox Laboratories, Crumlin, UK) and PON1 activity by using 4-Nitrophenyl acetate [53]. Triglicerides were also measured using an analyser (Olympus A 400, Tokyo, Japan) and following the manufacturer’s instructions. The concentration of albumin was measured by commercial reagents (Beckman Coulter, OSR6102). All assays showed an intra- and interassay imprecision lower than 10%. The low imprecision found in the assays resulted in using one single measurement and not replicates.

Expanded Disability Status Scale (EDSS): This scale is used to assess functional disability in multiple sclerosis patients [45]. It is an ordinal scale based on a neurological examination for the eight functional systems (pyramidal, cerebellar, brainstem, mental, sensory, visual, bowel and bladder), as well as an assessment of walking capacity, providing a disability index between 0 and 10, 0 being understood as having normal health and 10 representing death from MS.

### 2.6. Ethical Considerations

The study was developed in accordance with the Declaration of Helsinki [54], once the protocol was approved by the Human Research Ethics Committee of the Experimental Research Ethics Committee of the University of Valencia (procedure number H1512345043343). The study protocol was approved by the Ethics Committee of the Universidad de Valencia.

### 2.7. Data Analysis

Statistical analysis was performed with the SPSS v.23 tool (IBM Corporation, Armonk, NY, USA). The first step aimed to estimate the distribution of the variables investigated through statistical methods for the assessment of normality, including the Kolmogorov–Smirnov test. This analysis demonstrated the non-normal distribution of variables. Therefore, a non-parametrical test was used to assess the differences between groups. Categorical data were analysed with a chi-squared test. Finally, a two-tailed Spearman’s test was used for the correlation analysis. A *p*-value below 0.05 was considered significant. Data are presented as the mean ± standard deviation, or the number of patients and percentage.

## 3. Results

The final sample of this study included 51 patients diagnosed with MS, of which 72.5% had RRMS and 27.5% had SPMS. The patients in the study were between 22 and 70 years of age, with percentages of both sexes (70.6% women) representing what is currently regarded for the disease [55,56]. These socio-demographic variables exhibited no significant differences between the groups (Table 1).

In the same way, clinical variables before the intervention show no significant differences between the groups at baseline (Table 2).

**Figure 4:**
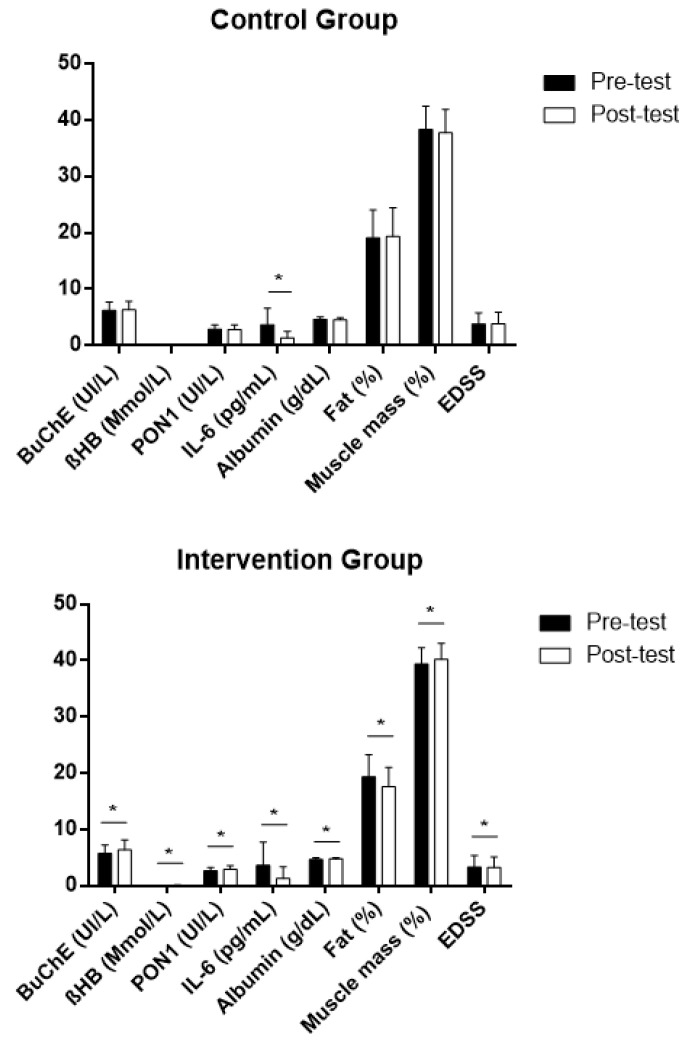
Intragroup differences in the study variables after intervention (post-test) expressed in average values ± standard deviation, both in the intervention and the control groups. BuChE: butyrylcholinesterase; βHB: β-hydroxybutyrate; PON1: paraoxonase 1; IL-6: interleukin 6; Fat (%): body fat percentage; Muscle mass (%): body muscle percentage; EDSS: Expanded Disability Status Scale; Z: Wilcoxon signed-rank test. * Statistically significant differences, *p* < 0.05.

The pre–post intragroup comparison indicated a significant difference only for IL-6 in the control group, which decreased after 4 months of the study. However, significant differences could be found in the intervention group for the levels of BuChE in the blood that increased. Additionally, and as we have already reported in previous studies for this population, in the intervention group, βHB, PON 1 activity and albumin increased significantly in the blood [57], whereas IL-6 decreased significantly, as did EDSS (representing an improvement in functional capacity) [58]. Moreover, the percentage of fat, which was within the values of normality for the range of age in the study population for both groups (19.10 ± 4.96% in the control group and 19.34 ± 3.98% in the intervention group), only decreased in the group that received the intervention (Figure 4). This decrease in the levels of fat mass could be related to a significant improvement after an intervention focusing on lean mass, which was observed in a previous study in our laboratory for the same group (from 38.01 ± 4.02% to 41.10 ± 2.81%) and which did not occur in the control group [57]. No patient showed a relapse that could have influenced the levels of IL-6 over the 6-month duration of the intervention.

On the other hand, after the intervention, there were positive correlations between the levels of BuChE, fat mass percentages, levels of triglycerides in the blood and PON1 activity. However, these correlations did not occur in the control group (Table 3).

## 4. Discussion

MS is a disease of an inflammatory nature which is characterised by the progressive loss of muscle associated with an increase in fat [6]. Conversely, the antioxidant EGCG has been shown to decrease inflammation and body fat percentage [59]. In this same sense, increases in ketone bodies in the blood are related to body composition changes characterised by weight loss [60] and an increase in muscle mass; thus, weight loss is mainly due to fat loss [49] along with a decrease in inflammation [36]. Coinciding with these results, after administering EGCG, together with coconut oil (which led to a significant increase in βHB in serum), we observed that there was indeed a significant decrease in the fat percentage which was within normal levels for healthy people, but also with an increase in muscle mass, which did not occur in the control group in our study. On the other hand, there was a significant decrease in the levels of cytokine IL-6. However, IL-6 levels in the blood also decreased in the control group, which could be explained by the microglial-level effects of the Mediterranean diet administered in both groups. It has recently been evidenced that oligodendrocytes exposed to oxidative stress in vitro have the potential to activate cells of the microglia, especially by means of IL-6, making it a protagonist of the disease’s inflammation process [61]. IL-6 activates astrocytes and microglia, regulating the expression of neuropeptides after neuronal injury, for example, in neurodegenerative diseases [62]. In this sense, it has been shown that following a Mediterranean diet decreases the production of proinflammatory cytokines produced in the microglia, and especially IL-6 [63] observed on a peripheral level [64]. The anti-inflammatory effect of this diet is due to its composition, characterised by a low intake of saturated fat from butter, full-fat milk and red meat, a high intake of monounsaturated fat found in olive oil, an adequate intake of omega-6 versus omega-3 polyunsaturated fatty acids from fish, shellfish and nuts, low contents of protein obtained from red meat, high quantities of fruit, vegetables, olive oil, herbs and spices, and high quantities of fibre found in products of plant origin, such as vegetables, fruit, whole grains, pulses and nuts.

However, despite the possible anti-inflammatory effect of the basal diet for both groups being determined by a decrease in IL-6, in our study, albumin only increased in the intervention group. This result could minimise the effect of the diet, because albumin is related to the diet because it is a marker for nutritional state, but also its increase indicates a decrease in inflammatory state [65]; therefore, the levels are inversely correlated with inflammation markers such as IL-6 [38].

Regarding anthropometric changes, possibly as a result of improvements with a decrease in the percentage of fat in the intervention group, a significant increase in PON1 activity in serum was observed (which was not observed in the control group), in line with what has been published by other authors after treatment with antioxidants [65]. Nonetheless, regarding BuChE, despite it being an enzyme related to the pathogenesis of the disease [29,30], a significant increase was observed only in the intervention group of our study. This could be based on the activity of the enzyme in lipid metabolism, as already observed by other authors [66]. In this sense, BuChE KO mice become obese with a high-fat diet [67]; BuChE is associated with the levels of lipoproteins in the blood, taking on a role in its metabolism and correlating it with its levels in pathologies, such as metabolic syndrome [68]. In this sense, this role in lipid metabolism could explain, on the one hand, the positive correlation with PON1 activity observed after our intervention, because both molecules show this lipolytic activity [45,69]; on the other hand, the positive correlation with fat levels in this same group could mean that the higher the fat percentage, the higher the BuChE activity. Furthermore, we also observed a positive correlation with the levels of triglycerides in the blood, as already observed in patients with cardiac risk [66]. The increase in BuChE activity in our study, as well as the positive correlation with these lipoproteins, would not be a result of inflammation derived from high levels of triglycerides [70], but due to an increase in triglycerides in the blood from coconut oil (which contains high levels of medium-chain triglycerides), which possibly reflects an increase in lipolytic activity, that is linked to the increase in that was ketone bodies (βHB) observed in the individuals.

On the other hand, the changes achieved at an anthropometric level and in serum markers may also be correlated at the central level, because we could observe an improvement in functional capacity directly linked to the presence of fatigue associated, in turn, to the BuChE activity in the CNS [28]. These results could confirm the clinical benefits after anthropometric improvements that could lead to an increase in BuChE in the blood, thus highlighting the role that this enzyme has both on a central and peripheral level, as already suggested by other authors [71].

Finally, these results need to be confirmed because this study was somewhat limited. These limitations include a small sample, and the measurement of other inflammation markers related to the disease need to be considered, among which the following could of interest: tumour necrosis factor (TNF) or C-reactive protein (CRP), and the anti-inflammatory cytokine IL-10. In addition, it would be interesting to determine not only the impact of the intervention of a peripheral level, but also in terms of the CNS, with the intention of establishing the possible relationship of BuChE with ACh or remyelination, which has recently been observed after following a ketogenic diet [72,73]. In addition, we propose the assessment of the behaviour of BuChE after this kind of treatment in the future, in terms of age, gender, and time after disease diagnosis. 

## 5. Conclusions

Administering EGCG along with an increase in ketone bodies in the blood after coconut oil intake causes a decrease in body fat, and as a consequence, it seems to improve the inflammation and oxidation levels of MS patients. In relation to BuChE, this enzyme seems to have a role in increasing the lipolytic activity; its levels increase after treatment, and at the same time, it is positively correlated with fat percentage, levels of triglycerides in the blood, and PON1 activity.

## Figures and Tables

**Table 1:** Socio-demographic characteristics of the population of the study.

		CG (*N* = 24)	IG (*N* = 27)	Total (*N* = 51)		
		Freq (%)	Freq (%)	Freq (%)	Chi^2^	*p*
Gender	Women	14 (58.3)	22 (81.5)	36 (70.6)	3.279	0.07
	Men	10 (41.7)	5 (18.5)	15 (29.4)		
MS Type	Relapsing–remitting	17 (70.8)	20 (74.1)	37 (72.5)	0.067	0.80
	Secondary progressive	7 (29.2)	7 (25.9)	14 (27.5)		
		Mean ± SD	Mean ± SD	Mean ± SD	Z	*p*
Age (years)		49.83 ± 12.42	44.56 ± 11.27	47.04 ± 12.00	−1.558	0.12
Diagnosis time (years)		14.21 ± 8.40	11.89 ± 9.74	12.98 ± 9.12	−1.418	0.16

CG: control group; IG: intervention group; MS: multiple sclerosis; SD: standard deviation; Z: Mann–Whitney U Test.

**Table 2:** Clinical characteristics of the population of the study before intervention (pre-test).

	CG (*N* = 24)	IG (*N* = 27)		
Variable	Mean ± SD	Mean ± SD	Z	*p*
BuChE (UI/L)	6.23 ± 1.48	5.79 ± 1.48	−1.231	0.22
βHB (Mmol/L)	0.05 ± 0.02	0.06 ± 0.04	−0.932	0.35
PON1 (UI/L)	2.88 ± 0.77	2.67 ± 0.62	−0.832	0.41
IL-6 (pg/mL)	3.67 ± 2.94	3.66 ± 4.10	−0.705	0.48
Albumin (g/dL)	4.66 ± 0.41	4.69 ± 0.29	−0.414	0.679
Fat (%)	19.10 ± 4.96	19.34 ± 3.98	−0.370	0.71
Muscle mass (%)	38.38 ± 4.15	39.39 ± 2.88	−0.547	0.584
Weight	70.44 ± 18,13	68,63 ± 13,56	−0.245	0.806
BMI	25.72 ± 6.01	25.92 ± 5.29	−0.142	0.887
EDSS	3.80 ± 2.00	3.37 ± 2.03	−0.780	0.435

BuChE: butyrylcholinesterase; βHB: β-hydroxybutyrate; CG: control group; IG: intervention group; PON1: paraoxonase 1; IL-6: interleukin 6; Fat (%): body fat percentage; Muscle mass (%): body muscle percentage; BMI: body mass index; EDSS: Expanded Disability Status Scale; SD: standard deviation; Z: Mann–Whitney U Test.

**Table 3:** Correlation in the study between BuChE and the other variables after intervention (post-test), both in the intervention and the control group.

			IG (*N* = 27)	
		Fat (%)	Triglycerides (mg/dL)	PON1 (UI/L)
BuChE (Mmol/L)	Coef	0.752	0.522	0.448
	*p*	0.000 **	0.006 **	0.022 *
		**CG (*N* = 24)**		
BuChE (Mmol/L)	Coef	0.235	0.299	0.186
	*p*	0.364	0.244	0.475

IG: intervention group; CG: control group; Fat (%): body fat percentage; PON1: paraoxonase 1; BuChE: butyrylcholinesterase; Coef: Spearman’s rank correlation coefficient; * statistically significant differences, *p* < 0.05; ** statistically significant differences, *p* < 0.01.

## Data Availability

Not applicable.
